# Editorial: Psychiatrization of society

**DOI:** 10.3389/fsoc.2023.1258264

**Published:** 2023-10-11

**Authors:** Timo Beeker, Anna Witeska-Młynarczyk, Sanne te Meerman, China Mills

**Affiliations:** ^1^Department of Psychiatry and Psychotherapy, Brandenburg Medical School, Immanuel Klinik Rüdersdorf, Rüdersdorf, Germany; ^2^Institute of Sociology, Maria Curie-Skłodowska University, Lublin, Poland; ^3^Faculty of Behavioural and Social Sciences, University of Groningen, Groningen, Netherlands; ^4^Health Services Research and Management Division, School of Health Sciences, City University of London, London, United Kingdom

**Keywords:** psychiatrization, medicalization, overdiagnosis, health system research, psychiatric epidemiology, psychiatric ethics, pharmaceuticalization

Worldwide, there have been consistently high or even rising incidences of people classified as mentally ill (Bloom et al., 2011; World Health Organization, 2019), paired with increasing mental healthcare service utilization over the last decades (Lipson et al., 2019; Olfson et al., 2019). While psychiatric institutions have been successively expanding, psychiatric knowledge has become increasingly dispersed and globalized, making psychiatric vocabularies and classificatory systems widely available, shaping increasing areas of life, creating powerful markets for therapeutic services of all kinds, and impacting how we understand ourselves and others. This process can be described as the psychiatrization of society (Beeker et al., 2021). Psychiatrization is highly complex, diverse, and global, although it takes different forms in different contexts, involves various actors with largely diverging motives, and is part of a wider assemblage of the psy-disciplines.

The effects of psychiatrization are vast and varied. Individuals or groups might well benefit from aspects of psychiatrization, as the growing mental healthcare system can also increase accessibility of services that are subjectively helpful (Lancet Global Mental Health Group et al., 2007; Thornicroft et al., 2017). In this context, psychiatric diagnosis may essentially determine which quality and quantity of support is available for people in distress. Yet psychiatrization can be potentially harmful to individuals and to public healthcare, e.g., through overdiagnosis and overtreatment (Moynihan et al., 2012), the psychological burden of being labeled (Livingston and Boyd, 2010), the epistemic injustice inherent in not valuing the knowledge of those with lived experience (Leblanc and Kinsella, 2016), and, in the Global North, exploding costs to meet the needs of the “worried well” (Wang et al., 2007). From a societal perspective, psychiatrization may further narrow down what is perceived as normal, diverse attention from the structural determinants of mental health and boost medical interventions which incite individual coping instead of encouraging long-term political solutions (Davies, 2017). In the Global South, where biomedical psychiatric practice is to a large degree exerted by trained non-specialists, psychiatrization could lead to excessive diagnosis and prescription of medication with little monitoring (Mills, 2014) while the expansion of westernized, colonially informed psychiatry risks undermining local support systems (Davar, 2014).

Given the rich body of research on medicalization (Zola, 1972; Illich, 1974; Conrad, 1992, 2005) with seminal publications going back to the 1970s, the question is legitimate why a whole Research Topic should be dedicated to psychiatrization, which might be perceived as just one special branch of medicalization among many others. It seems safe to say that there are more than enough medical specialties in which overdiagnosis and overtreatment are posing significant problems to patients, clinicians and public finance. So why should we speak of psychiatrization but not, for example, of an “urologization” of society to criticize the widespread overdiagnosis of prostate cancer (Vickers et al., 2023)?

The above listed effects of psychiatrization already indicate the reason: There might be much more at stake than harm through sub-optimal treatment decisions and the irresponsible use of taxpayers' money. As several contributions to this Research Topic compellingly show, psychiatry has the power to shape large parts of modern societies and is increasingly used to handle its discontents. By defining, for example, which kinds of human suffering should be understood as individual pathologies, psychiatric diagnosis ultimately becomes an important terrain of negotiation for fundamental questions such as how we expect ourselves and other people to be or in which kind of society we want to live. And in an ironic contrast to the scope of potential consequences of psychiatric diagnosis, there might be no other medical branch where it is so easy to invent new disease entities or to expand criteria for diagnosis, due to the notorious lack of objective correlates of psychiatric disorders, which pushes the doors wide open for the inflationary use of psychiatric concepts and treatments.

Apart from the more obvious large-scale impacts that are mentioned above, psychiatrization may become tangible in a multitude of more subtle phenomena, for instance in science publishing: when we set up this Research Topic, our aim was to motivate scientific contributions from a broad array of fields, following our understanding of psychiatrization as being an interdisciplinary phenomenon. Clearly, we wished for academic psychiatry to play a main role among the contributing disciplines. However, when we asked Frontiers in Psychiatry to co-host our Research Topic with Frontiers in Sociology, which is actually a very common design in the Frontiers' universe, our query was denied with the brief notice that “*psychiatrization of society*” as a topic was not of interest for the journal because it would not fit well with its aims. But when mainstream psychiatry fails to understand that debates on its role in society are relevant to its very nature, this seems to be a highly problematic self-conception. It also begs the question of how psychiatry as a practical and scientific discipline can possibly be trusted to responsibly manage its various and often controversial impacts on society, when it does not acknowledge its situatedness within the realm of the social. Adding to that, a scientific discourse that becomes hermetic toward the perspectives from other disciplines risks to lose the essential openness that characterizes every true scientific endeavor.

However, we were very happy to receive many valuable contributions. In their totality, they may help to shed a light on how psychiatrization can be conceptualized, how it manifests in different terrains, its effects on individuals and societies, and strategies to counter psychiatrization. Despite that, any kind of heuristics risks falling short of their variety and complexity, the articles are presented in chapters that reflect the *theoretical, practical*, and *political* dimensions of psychiatrization, with a special emphasis on the lively debate about the *psychiatrization of childhood* (Beeker et al., 2020).

## Theorizing psychiatrization

In Beeker et al., the authors present a working definition of psychiatrization as a “complex process of interaction between individuals, society, and psychiatry through which psychiatric institutions, knowledge, and practices affect an increasing number of people, shape more and more areas of life, and further psychiatry's importance in society as a whole”. As a starting point for further research, the authors suggest a basic model of psychiatrization. This model takes into account that psychiatrization is not exclusively caused in a top-down-way by organized psychiatrists or the pharmaceutical industry, but quite frequently co-produced by top-down and bottom-up-interactions. The latter may originate from a demand for support, recognition or explanations by patients, consumers and ordinary citizens without professional ties to the healthcare system.

In direct reply, Haslam et al. compare psychiatrization with their seminal idea of “concept creep” that was first described by Haslam in 2016 (Haslam, 2016). Concept creep refers to the gradual expansion of harm-related concepts such as addiction, prejudice, or bullying, that were semantically re-shaped over the last decades to include an increasingly wide range of phenomena. The authors show that their original conception of “vertical” vs. “horizontal creep” can be applied fittingly to diagnostic inflation in its twofold meaning of relaxation of diagnostic criteria respectively creation of new diagnostic entities. They suggest considering that psychiatrization may be embedded in the same cultural dynamics as concept creep, which is a growing sensitivity to harm with a tendency to its amplification. Thus, concept creep and psychiatrization may have similar ambivalent effects by drawing attention to neglected harms or illnesses but inflating also minor harms in a problematic way at the same time.

Demke provides a close critical reading of the influential vulnerability-stress-model, finding that while the model appears to integrate social dimensions of mental health, it also perpetuates a medicalised view of faulty individuals. The author questions the very idea of inherent vulnerability—with its potential to divert “attention from the gravity of actual wounds, which would have to be taken seriously in order to open up empowering avenues such as fighting for one's rights and against discrimination, victimization and other grievances that are known to make people unwell”. She situates the model, as it emerged in the 1970s, during a period of fundamental critique of psychiatric theory and practice, showing how such critique can be integrated into psychiatry while “allowing for a continued reliance on core elements of the medical model such as the focus on the inherently deficient individual and mandatory pharmaceutical intervention”.

Topor et al. describe how “recovery” evolved from a radical concept questioning the core of psychiatric practice and knowledge to an idea that has become increasingly psychiatrized itself. Starting as a concept that emphasized the social character of mental health and promoting hope for individuals that the use of psychiatric services could be left behind once and for all, the concept of recovery underwent a transition during which it became gradually individualized and detached from the social. This streamlined notion of recovery finally became even integrated into the psychiatric services, where it did not mean much more than a never-ending personal journey. In contrast to such a shallow, de-socialized view, the authors advocate the reappropriation of the concept of recovery as a “deeply social, unique, and shared process in which our living conditions, material surroundings, social relations and sense of self evolve”.

Russo shifts the focus of this volume by urging researchers concerned with the concept of psychiatrization to clearly define their position in relation to their field of study. She poses a critical question on how to prevent the (re)psychiatrization of our own research work. The author argues from a mad studies' perspective that psychiatrization is not something separate from us as researchers; rather, it is an integral part of the knowledge production on mental health and distress in which we are actively involved. The author encourages us to examine our perspectives, research ethics, and the manner in which we communicate our findings. Her text can be interpreted as both a manifesto and a call for a candid debate about the potential for enacting transformative research within the existing structures of knowledge production. When viewed as a personal issue, a political matter, and a strategy for de-psychiatrizing our own research, it delves into the epistemological and ethical foundations that underlie the social production of knowledge. Specifically, the author advocates for a radical shift toward de-psychiatrization in our work and invites us to actively participate in this crucial endeavor.

## The psychiatrization of childhood

Witeska-Młynarczyk suggests examining the adoption practices in contemporary Poland as a part of larger processes of psychiatrization. She provides an ethnographic account of what she calls “the advancing psychiatrization of kinning”. This phenomenon occurs at the intersection of family and social policies as the medicalization, and psychologization of familial relationships. Taking a diachronic perspective, the author offers a portrayal of the adoption network and its functioning. She perceives it as facilitating the “privatization of the social problem” and working toward individualizing the responsibility for its resolution. To describe the ways in which the network of public institutions, relying on psy-knowledge, assesses children and prospective parents for adoption, as well as educates future parents about the therapeutic role their future family should play, Witeska-Młynarczyk employs the concept of “biopolitical bureaucracy” (Nissen and Bech Risør, 2018). She also introduces the notion of “invisible disabilities” (Blum, 2015) to discuss the range of anxieties, self-doubts, and intense emotions generated within this context set in motion by the state. Once adoption is legalized, the new family is compelled to embark on a solitary “diagnostic journey”, bearing the full financial and emotional responsibility while completely absolving the state of its role. This is coupled with a growing interest of psychiatry in mental health of adopted young people, as well as in adoptive family as such.

Batstra et al. argue that to avoid unnecessary psychiatrization, schools potentially need to be a primary target as teachers are often the first to instigate a psychiatric classification. However, reification is a pervasive problem. Reification refers to the process of presenting behavioral descriptions from the DSM, like ADHD, as disease entities. A major driver of reification is for instance the widely made “ecological fallacy” which means that very small average differences like slower brain maturation in groups with an ADHD classification are presented as if everyone with a classification displays such a pattern of brain growth. Reification is at odds with the goal of inclusive education, because the perception of unwanted behaviors as caused by medical entities entails the (psychiatric) adjustment of children to make them fit in. The authors contrast this with a more community-based view of disability that holds the position that it is not disabilities but barriers in society that cause exclusion. The authors argue that for such a community-based approach to be successful however, a small but pervasive perceptual shift might be necessary. Rather than singling out children as having special needs to be addressed, the focus can instead be placed on teachers who -as an inherent part of the professionalized socialization- will always need some degree of special needs to do their work. Hence, we should no longer be speaking about children with special needs but about teachers with special needs.

However, the many challenges that need to be faced when moving away from an individualized narrative become clear in the study by Honkasilta and Koutsoklenis. The authors debunk the feeble scientific basis of a classification like ADHD, for instance by looking at the ambiguous, overlapping, and rather arbitrary criteria, obviously informed by contemporary norms and societal values and changing from one version of the DSM to the next, without any real scientific rationale. However, despite the weak scientific base, the authors reveal how deeply engrained classifications like ADHD have become. They may serve as legal entities, deciding who gets additional services and goods but a classification may also provide a moral excuse for misbehavior and may even exempt from legal liability. Likewise, classifications can be instrumental for parents and children themselves as tools to evoke understanding and compassion. Some may feel empowered by classification such as ADHD in an attempt to embrace their alleged “differently wired brains”. At the same, classifications can remove agency, helped by the DSM discourse suggesting children are “unable” rather than unwilling to perform certain behaviors. Eventually, the pseudo-scientific discourse surrounding classification seems to create a reality rather than describe it, and unfortunately a reality that might severely restrain ways of being normal or even ways of being in general.

## Psychiatrization and medical practice

van Dijk et al. present a qualitative study on how general practitioners (GPs) in the Netherlands dealt with sadness complaints of young adults. Based on 13 interviews, a typology of GPs was developed. GPs who tended to a fast referral to specialist care were usually motivated by personal concern for their patients, by pragmatical reasons or by feelings of incompetence when confronted with seemingly psychiatric conditions. Sadness complaints, thus, were transferred quite easily into a medical condition. GPs who felt well prepared to recognize and treat psychiatric disorders themselves also tended to low-threshold diagnosis and pharmaceutical treatment in primary care. Only GPs who acknowledged that their responsibility may often transcend pure medical problems and, in consequence, saw themselves as partners to discuss the more or less existential questions of life, were inclined to non-psychiatrizing interventions such as watchful waiting. This result emphasizes that only those practitioners who are willing to set the biomedical framework aside in favor of true human encounter may be able to offer support in a non-psychiatrizing way.

In a similar vein, Beeker explores how psychiatrization may emerge from mental healthcare settings. The author focusses on the micro-level by analyzing two prototypical cases of patients coming to the emergency department of a general hospital to receive help in an initially undefined situation. The cases illustrate why decisions whether to label and treat a certain condition as a “mental disorder” or not, can be highly difficult for practitioners, especially in cases where the (health) concerns are rather moderate, and clearly associated with common life problems. However, psychiatrist's decisions may be largely biased in favor of psychiatrization by a wide array of top-down-drivers on the one hand, among which clinical routines, the vagueness of classificatory systems, the necessity of diagnosis for reimbursement of any kind of support and professionals' striving for the reduction of legal risks. On the other hand, also bottom-up mechanisms such as help-seekers' expectations and understandings of their own problems, that may be shaped by soft cultural factors or prior treatment-experiences of friends and family, may play a crucial role when negotiating the accurate interpretation of a situation of crisis.

Baumgardt and Weinmann forward the use of Crisis Theory as a less pathologizing and more normalizing approach to provide help in situations such as those witnessed by the emergency department. The authors discuss Crisis Theory against the background of the widely adopted but severely flawed medical nosology of the DSM and go on to discuss the stress-vulnerability model that was successively introduced to counter some of the limitations of the biomedical approach. Unfortunately, misapplication of the model again placed biological factors at the centerfold. Crisis Theory offers an alternative heuristic approach for understanding the nature and development of mental distress but is seldom explored to its full capacity. The authors discuss several misconceptions and problems that may hamper the adoption of Crisis Theory like it's supposed unsuitability to tackle more severe problems of people with an alleged biological disposition for mental illness–which psychiatry assumes to be different from those who experience a psychological, stress-related crisis. However, the authors clarify how, regardless of the alleged biological or stress-related nature of the problems, Crisis Theory can bring many improvements to the status quo, particularly by combining it with a system-oriented approach.

von Peter et al. explore if Open Dialogue (OD) has the potential to offer psychosocial support in a significantly less or even non-psychiatrizing way. OD was initially developed in Finland in the 1980 for patients with acute psychosis and from then on applied in more than 30 countries. Being essentially a kind of home-treatment with systemic background, it offers multi-professional, and needs-oriented support, nowadays also for users with various kinds of mental distress. As a core element, regular network meetings with the service users and their private or professional environments provide an opportunity to develop a shared understanding of the current crisis, and to make joint decisions for the further course of action. While previous research has shown that OD can limit the use of neuroleptics and decrease the use of psychiatric services, the authors explore the inner logic of OD for further potential for de-psychiatrization. They suggest that OD's tendency to encourage the use of everyday terms instead of the psychiatric idiom, together with a dialogical, polyphonic process of meaning making can be “breaking the interpretative sovereignty of psychiatric language” and concepts. In this polyphonic process, psychiatrists and other health professionals become only individual voices among many others. Instead of communicating psy-knowledge in a top-down way, their new role is to facilitate the dialogical quest for a mutual understanding and for adequate, by far not only medical help. In sum, OD could be a promising means to offer a different, less-psychiatrizing kind of support, shifting the emphasis from individualizing medical thinking toward a more social model of crisis and help.

## Politics of psychiatrization

Logan and Karter analyze psychiatrization as a kind of “ontological politics”, that imposes narrow interpretative limits on states of difference and distress and tends to exclude other possible meanings. By doing so, psychiatrization may function as a tool of disciplinary control of any kind of resistance against hegemonic norms and institutions of gendered or racialized oppression in domestic or international contexts. The authors exemplify their hypothesis with a close look on consumer/survivor/ex-patient and psychosocial disability movements in the Global South. They demonstrate how psychiatrization may thwart activists' original aims of transforming both the mental healthcare system and the political weight of mental distress, and advocate for understanding at least some mental suffering as a materialization of discontent with oppressive political or socioeconomic conditions.

In a thorough Marxist analysis of the mental health system, Moncrieff argues that the concept of mental illness (understood as an individual medical problem) plays a strategic role in contemporary societies. Specifically, it works to obscure the failings of the neoliberal economic system. The author takes the United Kingdom as a case study to explain how the public mental health system has evolved alongside capitalism, catering to the regulatory needs of the labor market. Adopting a synchronic approach, she unravels trends typical of the capitalist system in general, with a particular focus on the neoliberal system. In this context, large segments of the post-industrial population are marginalized and categorized as mental patients. Indicators of the changing structure of capitalism include the widespread consumption of antidepressants and the increasing psychiatric diagnoses. The author does not limit herself to a critical analysis but also emphasizes the need for political change based on a radical rejection of the medicalization of “so-called” mental health problems.

Schumann et al. discuss Psychiatry's relation to right-wing extremism which they view as an example of undue top-down psychiatrization. For instance, they criticize the superficial tendency to focus on psychological vulnerabilities and social risk factors such as bad peer influence leading up to right-wing extremism. The authors argue that this narrow focus may lead to a predominantly individualized psychiatric gaze on right wing beliefs that are often better understood as a response of more complex factors such as societal conflict, economic uncertainty and societal processes of individualization and anomie. Furthermore, due to this narrow focus other areas of interests may be overlooked. For example, more conceptual understanding of the complex interplay between individual and social factors is needed, as well as a more practical orientation on the challenges that patients and staff may face when working with patients with right-wing tendencies.

## Conclusion: the way ahead

The heterogeneity of the papers included in this collection demonstrates once more the complexity of psychiatrization as a field of research and gives a glimpse into the many different ways that psychiatric knowledge and practices may be engrained into contemporary societies. Further studies following different epistemologies and using different methodologies still seem necessary to get a clearer view on the scope, the origins, the mechanisms and the various impacts of psychiatrization—including its influence on the researchers themselves. However, despite the magnitude of this endeavor, a dash of optimism appears legitimate: as this collection shows, a critical interdisciplinary analysis of psychiatrization seems to be possible and worthwhile, especially when research is conceptualized as dialogical and multi-perspective.

Nevertheless, research alone cannot be an end in itself. The negative effects of psychiatrization are ubiquitous and significant, as constantly voiced by the user-/survivor-movement and underlined by this collection. Many creative ideas will be needed to build up measures of psychosocial support that are not psychiatrizing, but provide effective and sustainable help in situations of crisis. The articles of this Research Topic may offer valuable inspirations for winding back some of the harms of psychiatrization and to start doing what will most likely be inevitable on the long run: to move away from the individualized, medical perspective with its narrow confines toward a broader view that dares to re-contextualize and re-politicize human suffering.

## Author contributions

TB: Conceptualization, Writing—original draft, Writing—review and editing. AW-M: Writing—original draft, Writing—review and editing. SM: Writing—original draft, Writing—review and editing. CM: Writing—original draft, Writing—review and editing.
